# Removal of Flotation Collector *O*-Isopropyl-*N*-ethylthionocarbamate from Wastewater

**DOI:** 10.3390/molecules26216676

**Published:** 2021-11-04

**Authors:** Zhe Wang, Jun Yao, Mojca Bavcon Kralj, Darko Dolenc, Polonca Trebše

**Affiliations:** 1 Faculty of Chemistry and Chemical Technology, University of Ljubljana, Večna pot 113, 1000 Ljubljana, Slovenia; wangzhe19900512@163.com (Z.W.); darko.dolenc@fkkt.uni-lj.si (D.D.); 2 School of Energy and Environmental Engineering, University of Science and Technology Beijing, Xueyuan Road 30, Beijing 100083, China; 3 School of Water Resources and Environmental Engineering, China University of Geosciences Beijing, Xueyuan Road 26, Beijing 100083, China; yaojun0804@live.cn; 4 Faculty of Heath Sciences, University of Ljubljana, Zdravstvena pot 5, 1000 Ljubljana, Slovenia; mojca.kralj@zf.uni-lj.si

**Keywords:** *O*-isopropyl-*N*-ethylthionocarbamate, *O*-isopropyl *N*-ethylcarbamate, iron salts, photocatalysis, hypochlorite, Fenton reaction

## Abstract

Flotation collector *O*-isopropyl *N*-ethylthionocarbamate (IPETC) is widely used for separation of sulfide ores. Its removal from water by several oxidation processes was studied. Photocatalytic oxidation with air in the presence of iron salts, utilizing solar irradiation or artificial UV-A light is very efficient. Oxidation leads through the formation of *O*-isopropyl *N*-ethylcarbamate and several other reaction intermediates to total decomposition of organic compound in the final stage in 1 day. Similar results were obtained with a Fenton type oxidation with hydrogen peroxide and iron salts. Treatment with sodium hypochlorite yields mainly *O*-isopropyl *N*-ethylcarbamate. The formation of this compound in wastewaters can be of concern, since simple alkyl carbamates are cancer suspect agents.

## 1. Introduction

*O*-isopropyl *N*-ethylthionocarbamate (IPETC) is widely used as a flotation collector for sulfide ores, particularly copper and silver ones [1]. Leakage of this compound into wastewater after ore separation is unavoidable. The water is frequently discharged directly into the environment without any treatment [2]. It is known that several of residual sulfide mineral flotation collectors in flotation wastewaters even in low concentrations are toxic to water life. Serious environmental problems associated with the flotation reagents in water from mineral processing plants have been documented [3,4]. Although scientific papers dealing with the toxicity of IPETC are not available, it is considered as potential carcinogen and harmful to aquatic life with long lasting effects [5]. Related compounds where sulfur atom is replaced by oxygen, carbamates, are also known as potentially bioactive compounds. Although weakly toxic to mammals, [6] carbamates are highly toxic to some arthropods and *O*-aryl *N*-alkylcarbamates are used as insecticides. Several low molecular weight carbamates (ethyl urethane, methyl carbamate etc.) are considered as cancer suspect agents [7,8].

IPETC is a kind of nin-ionising collector of oily liquid, slightly soluble in water with different forms in different pH environments [2]. There is virtually no information in the literature concerning the behavior and stability of IPETC in the environment, as well as its toxicity properties. Research was conducted on the adsorption of IPECT on mineral surface [9,10,11], but the interaction mechanism in not well understood. Some research was also conducted on the microbiological degradation of IPETC under anaerobic conditions using anaerobic digester sludge [12,13]. It was found that the compound is degraded by mixed cultures in the presence of nitrate, Fe^3+^ and other electron acceptors. In another study, IPETC was subjected to aerobic biodegradation conditions and it was found to be poorly degradable [14]. 

Biodegradation is one of the most important processes for removal of organic pollutants in natural waters. However, this can be a very slow process and requires a quantity of microorganisms living under suitable conditions, as pH, appropriate level of oxygen, nutrients, absence of toxic substances, etc. In mineral separation wastewater this is rarely the case. These waters frequently contain heavy metals, acids, or alkalis and other toxic substances, which inhibit or prevent microorganisms from growing and functioning. In such a case, chemical treatment of wastewater may become necessary. Regardless of the type of treatment of polluted water, reaction intermediates emerge from the initial pollutants, which can also be harmful or even more toxic than the starting material [15,16]. The study of degradation pathways, including all important reaction intermediates and their properties is of prime importance. AOPs find suitable applicability in treatment of waters containing harmful chemicals. The advantage of those kind of methods is in in situ generation of strong oxidants, i.e., hydroxyl radicals and sulphate radicals, for the oxidation of organic pollutants [17]. Various photochemical combinations may be used such as UV/H_2_O_2_, UV/Fe^2+^, UV/H_2_O_2_/Fe^2+^, UV/TiO_2_, and others. The main advantage of UV AOPs is in better removal rates with both kind of radicals, i.e., hydroxyl and sulphate ones [18]. Photochemical AOPs are frequently used for the oxidation of organic pollutants in wastewater, but the combination of UV irradiation with hydrogen peroxide is usually more effective for specific micropollutants, such as diclofenac and mecoprop. Fenton process, on the other side, showed the fastest removal rate for phenol present in wastewater [19].

In this paper, we present the results of several approaches to the treatment of water containing IPETC, namely photodegradation with UV-A light in the presence of iron salts, Fenton reaction, and oxidation with hypochlorite.

## 2. Results and Discussion

### 2.1. Photooxidation in the Presence of Iron Salts

Effluents from mineral processing deposited into open containers or discharged into streams are subjected to solar irradiation. The first experiment performed on the solution of IPETC in water was irradiation in photochemical reaction with UV-A light. A 24 h irradiation with six 15 W lamps resulted in zero loss of the starting compound. Only after 1 week of irradiation a slight decrease in concentration of IPETC was noticed. Thus, it can be concluded that IPETC is photochemically stable under UV-A light. 

**Scheme 1:**
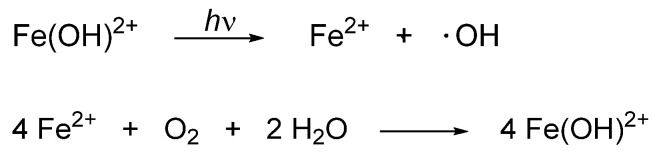
Simplified two-step reoxidation of Fe(II) complex.

In mining wastewaters, a presence of iron compounds is to be expected. Iron(III) compounds are well-known photocatalysts, producing hydroxyl radicals in water under visible or UV-A light [20,21,22,23]. Fe(II), formed in the photocatalytic reaction, can be regenerated with molecular oxygen. The predominant form of iron(III) in slightly acidic aqueous medium (such as the water solution of FeCl_3_) is Fe(OH)^2+^ and related species [24]. The process of reoxidation of Fe(II) is complex, however it can be simplified as shown in the Scheme 1.

**Figure 1:**
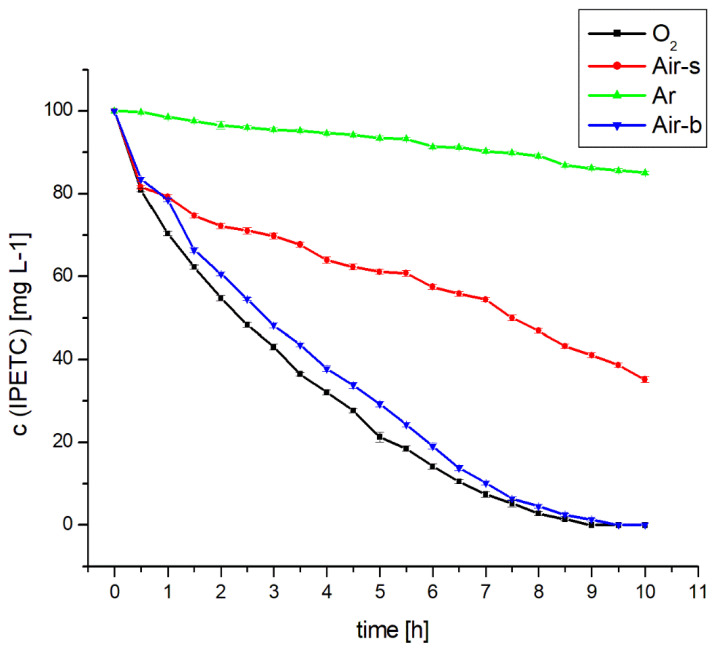
Decomposition of IPETC under UV-A light in the presence of FeCl_3_ in solutions with various concentrations of oxygen. Curve Ar: solution purged with argon. Air-s: saturated with air, no bubbling. Air-b: air bubbled continuously. O_2_: oxygen bubbled continuously.

Photochemical experiments with a water solution of IPETC and FeCl_3_ were carried out under various conditions. In the dark, a mixture of IPETC and FeCl_3_ exhibited no change in the concentration of IPETC across the period of several days. Under UV-A light, a decomposition of IPETC took place, with a rate depending on the concentration of oxygen (Figure 1).

Concentration of oxygen in air-saturated water at 20 °C and 97 kPa can be estimated to 0.28 mM [25]. The initial concentration of IPETC in these experiments (100 ppm) was 0.68 mM, which is several times higher than the concentration of oxygen. Thus, the amount of oxygen, dissolved in the reaction mixture is not enough to oxidize all IPETC, in particular because it is likely that more than 1 equivalent of oxygen is consumed in the oxidation. In Figure 1 (curve Air-s) it is clearly seen, that the initial rate of oxidation with air is fast, and after some time the rate slows down and becomes comparable to the rate of degradation in the absence of oxygen (curve Ar). When pure oxygen or air was bubbled continuously through the irradiated solution, the decomposition was fast, obeying 1st order kinetics in the beginning, changing after 70–80% conversion to a mixed order. The rate of oxidation is almost the same with pure oxygen as it is with air, indicating rapid reoxidation of Fe(II) with oxygen. When iron(III) sulfate was used instead of chloride, no significant change in the reaction rate was observed (not shown in Figure 1). With iron(II) sulfate no reaction was observed under argon. However the reaction commenced when argon was replaced by air.

**Scheme 2:**
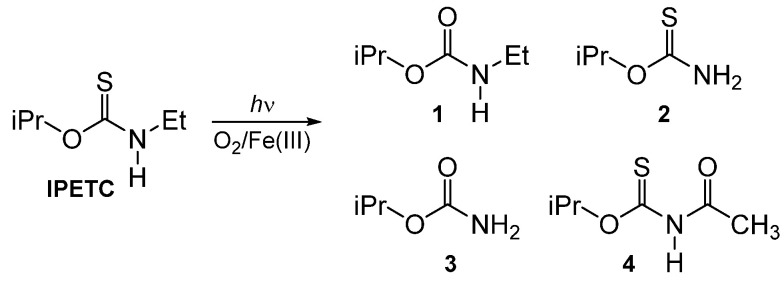
Transformation of IPETC under UV-A light in the presence of FeCl_3_ in solutions with various concentrations of oxygen.

The analysis of the reaction solution after the complete conversion of the starting IPETC revealed a formation of several products. HPLC with UV-DAD detector has detected one initial product, formed in significant amount (its peak in chromatogram is comparable to that of IPETC), which is slowly transformed to another compound on standing. According to its UV spectrum (λ_max_ = 262 nm, IPETC: λ_max_ = 244 nm), in the molecule of this first intermediate, the C=S group is most probably preserved, but it is lost in further reactions. Unfortunately this compound was found to be too unstable to be isolated and characterized. Further degradation leads to compounds which do not absorb UV and are difficult to determine by HPLC. GC and GC/MS analyses revealed several products, among which is *O*-isopropyl *N*-ethylcarbamate (**1**) was present in the highest amount. The identity of this compound was confirmed by independent synthesis [26]. Other, minor compounds are, according to their mass spectra, *O*-isopropyl thionocarbamate (**2**) and *O*-isopropyl carbamate (**3**, for both compounds, good matching with spectra in MS database was found). One of the minor products was tentatively assigned as *O*-isopropyl *N*-acetylthionocarbamate (**4**), all presented in Scheme 2.

All these compounds react further under irradiation. In sufficient time, all organic material is degraded. TOC measurements, performed in regular 8 h intervals show a slight increase of organic carbon in the first 16 h and a sharp drop after 24 h. Apparently, in the beginning, IPETC is transformed to other, still organic compounds, which are ultimately oxidized to CO_2_.

The exact degradation pathway could not be established. From the structure of the intermediates formed, the most probable pathway is that of several parallel reactions with hydroxyl radical taking place simultaneously. It can attack the C=S group, leading to the replacement of sulfur atom with oxygen, possibly via unstable *S*-oxide [27]. This is inferred from the formation of several compounds containing carbonyl group. Another site of attack is methylene hydrogen in ethyl group, which can be easily abstracted, leading to oxidation and/or dealkylation products [28,29,30,31,32].

To test the possibility of photodegradation of IPETC under natural conditions, a solution containing IPETC and iron was exposed to sunlight on a clear July day. After a whole day of irradiation, neither IPETC nor the products were found in the sample.

### 2.2. Fenton Type Oxidation with Iron Salts and Hydrogen Peroxide 

**Scheme 3:**
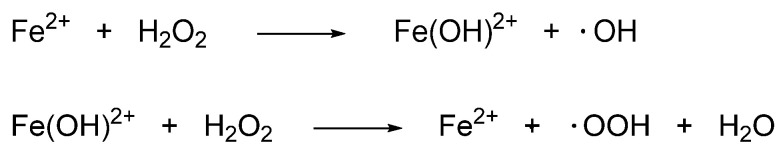
Reaction of iron salts with hydrogen peroxide and formation of peroxide radicals.

Another reaction system, producing OH radicals is a mixture of hydrogen peroxide and suitable redox metal salt, e.g., Fe, Cu, Ti and similar. Efficient and environmentally most acceptable are iron salts, which react with hydrogen peroxide according to the Scheme 3.

**Figure 2:**
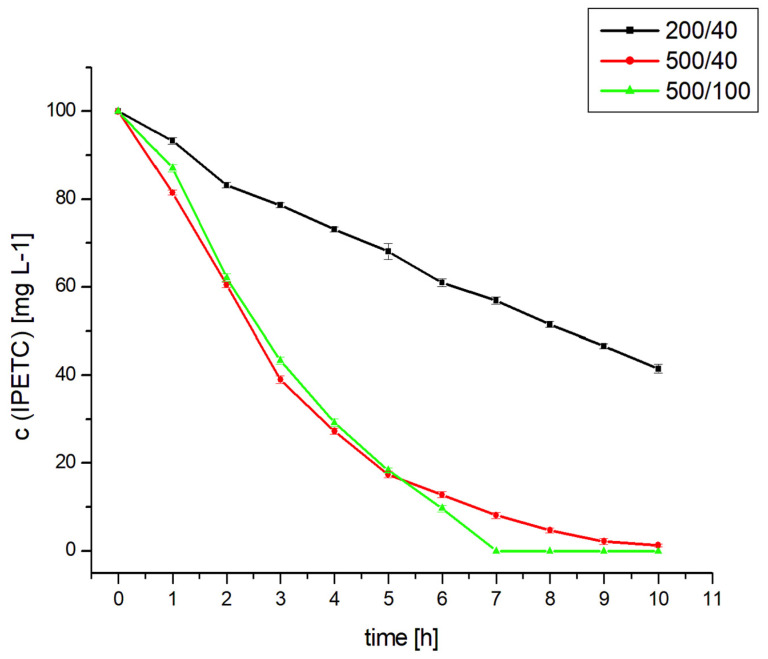
Decomposition of IPETC in FeCl_3_/H_2_O_2_ system. Numbers in the legend represent conc. of H_2_O_2_ and Fe in ppm.

Hydroxyl and hydroperoxyl radicals are produced, which react with organic substrates. The chemistry of these reactions largely resembles the processes in irradiated solutions of iron salts. Therefore it is not surprising that some of the the products appearing during Fenton reaction are the same as in photooxidation. However, there are differences since in Fenton system additionally contains hydroperoxyl radicals and hydrogen peroxide, which react in their own way. We have conducted experiments with several different concentrations of iron salts and hydrogen peroxide. In Figure 2 only few cases are presented for the sake of clarity. It can be clearly seen that the iron concentration has little or no effect of the reaction rate in selected concentration range. The rate of oxidation depends mainly on the concentration of hydrogen peroxide. The main product is again *O*-isopropyl *N*-ethylcarbamate (**1**).

### 2.3. Reaction with Sodium Hypochlorite

An alternative method for the removal of organic compounds from wastewater is treatment with strong oxidants, e.g., sodium or calcium hypochlorite [33,34]. Sulfur compounds are susceptible to oxidation, yielding various oxidation products. Sulfur atom is usually transformed to sulfate in the final stage [33]. 

**Figure 3:**
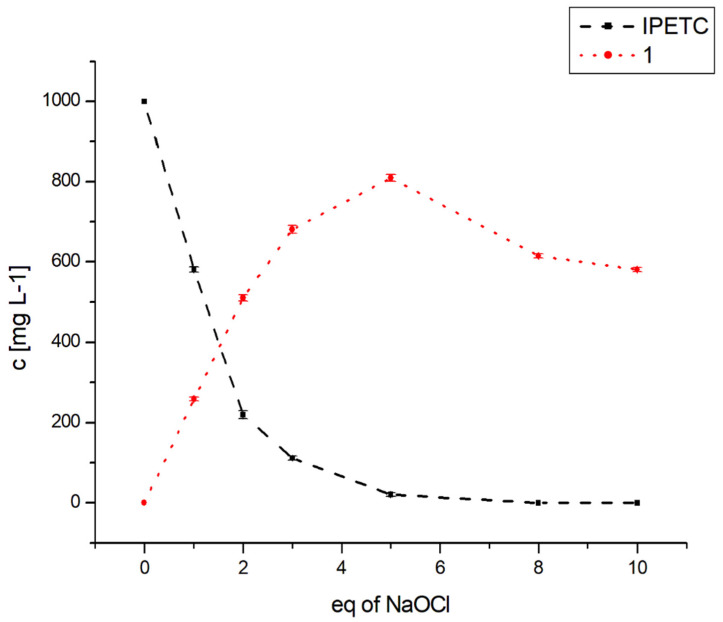
Degradation of IPETC with NaOCl in water at pH = 7.

Reactions of IPETC with NaOCl in phosphate buffer at pH 7 with various amounts of hypochlorite were performed. The reaction is fast, IPETC reacts within minutes. With small amounts of hypochlorite, two products were detected, the principal one being *O*-isopropyl *N*-ethylcarbamate, **1**. When increasing the amounts of hypochlorite added, the other compound disappeared. The amount of **1** decreased slightly on increasing amounts of hypochlorite (Figure 3).

**Scheme 4:**
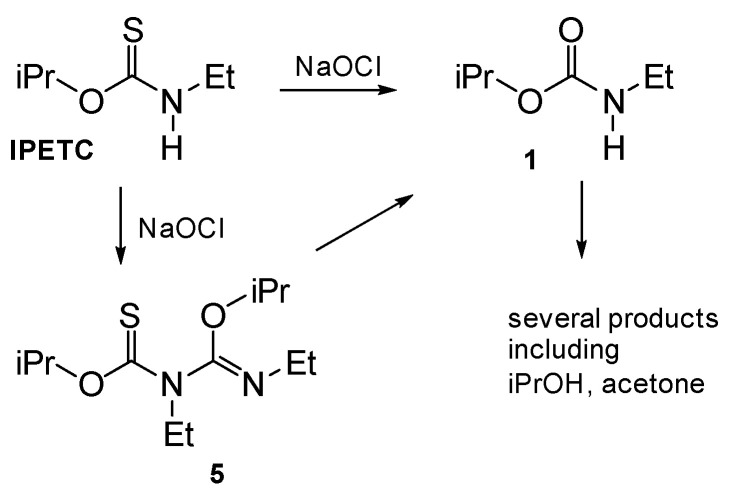
Transformation pathway of IPETC in the presence of NaOCl.

To elucidate the structure of the intermediate product, the reaction mixture obtained by the addition of two equivalents of NaOCl to IPETC was extracted with dichloromethane and the organic extract chromatographed on silica column. The structure of the compound was determined by several NMR techniques (^1^H and ^13^C-NMR, COSY, ^1^H-^13^C-HSQC, ^1^H-^13^C-HMBC, ^1^H-^15^N-HMBC), mass spectrum and HRMS measurement and was found to be isopropyl *N*-((ethylimino)(isopropoxy)methyl)-*N*-ethylthionocarbamate (**5**). Compound **5** is oxidized to **1** with additional amounts of hypochlorite (Scheme 4).

Carbamate **1** seems to be a relatively stable product of the oxidation of IPETC with hypochlorite in neutral medium. However it is slowly oxidized further with excess of hypochlorite, producing isopropanol, acetone and some other unidentified products.

Isopropyl ethylcarbamate (**1**) is a scarcely described compound and its toxicological properties are not known. It should be stressed that at least some related simple carbamates are cancer suspect compounds and this can possibly apply to **1** too. It can be formed from IPETC in any water, containing hypochlorite or related chlorine based disinfectants, including swimming pool and drinking water, as well as in other surface waters subjected to solar irradiation.

## 3. Materials and Methods

### 3.1. Chemicals

IPETC sample was a technical product (Tieling Flotation Reagents, Tieling city, Liaoning, China). Its purity was tested by ^1^H-NMR and GC and was established to be pure enough (less than 2% of impurities) to be used in the present study. Acetonitrile, dichloromethane, ethyl isocyanate (Aldrich) isopropanol, formic acid (Fluka), iron(III) chloride hexahydrate, iron(II) sulfate heptahydrate, sodium sulfite (Merck), hydrogen peroxide, 65% (Belinka, Ljubljana) were used without further purification. The concentration of sodium hypochlorite solution (Aldrich) was determined by iodometric titration prior to use. Deionized water was obtained from Millipore Milli-Q water system (Burlington, MA, USA).

### 3.2. Analyses of Reaction Mixtures

Quantitative analyses of reaction mixtures were preformed on HPLC-DAD instrument (Agilent, Santa Clara, CA, USA) with an external standard of IPTC or **1**.

For qualitative analyses of the reaction products, several instruments were used. GC and GC/MS analyses were carried out on HP 6890 instrument (Hewlett Packard, Palo Alto, CA, USA), equipped with HP5 (30 m × 0.32 mm i.d.) column and FID or quadrupole MS detector, respectively. Aqueous samples, containing iron salts and other inorganic material, for GC analyses were extracted with several portions of dichloromethane and concentrated under reduced pressure.

TOC analyses were performed on Shimadzu TOC5000A Total organic carbon analyzer (Shimadzu, Kyoto, Japan). Confirmation of structures of independently synthesysed products, HRMS measurements were performed on Agilent 6224 TOF LC/MS system (Agilent, Santa Clara, CA, USA), and NMR spectra were measured on Bruker Avance III 500 spectrometer (Bruker, Karlsruhe, Germany).

#### 3.2.1. HPLC Analyses

Samples from photochemical and Fenton degradation experiments were injected directly, without any preparation onto Agilent 1260 Infinity instrument (Agilent, Santa Clara, CA, USA), equipped with Phenomenex Luna 5μm C18 (250 × 4.6 mm) column and DAD photometric detector. Mobile phase consisted of deionized water, acidified with 0.1% of formic acid, and gradient grade acetonitrile (Aldrich). Calibration curves were measured in the range 0–1000 mg L^−1^ for IPETC and **1**.

#### 3.2.2. GC/MS Analysis

Reaction mixture (20 mL) from photooxidation, after all of the IPETC reacted, was extracted with 10 mL of dichloromethane in three portions, the extract concentrated under reduced pressure and subjected to GC/MS analysis. Several peaks were found and in some of them, the substances were identified according to their mass spectra (EI, *m/z* (%). Besides isopropyl ethylcarbamate (**1**) the following compounds were found: *O*-isopropyl thionocarbamate (**2**), *O*-isopropyl carbamate (**3**), and *O*-isopropyl *N*-acetylthionocarbamate (**4**). Details about mass spectra are presented in the Supplementary Materials section.

#### 3.2.3. Determination of Sulfate (Typical Experiment)

29.3 mg of IPETC and 250 mg of sodium hydrogen carbonate was dissolved in 50 mL of water. This solution was oxidized with 0.55 mL of 1.8 M NaOCl (5 equiv.) and left to stand 2 h at r.t. The solution was acidified with 2 mL of conc. HCl and sulfate determined gravimetrically as BaSO_4_. The mass of BaSO_4_ was 21.3 mg (0.458 mol/mol of IPETC).

### 3.3. Degradation Experiments

Photochemical experiments were carried out in photochemical reactor MLU 18 reactor (Photochemical Reactors Ltd., Surrey, UK) with six 15 W UV-A lamps (Sankyo Denki FL-15BLB).

#### 3.3.1. Photochemical Degradation Experiments

In total, 10 mL of a solution of IPETC (100 ppm, 0.68 mM) and FeCl_3_ × 6H_2_O (or other iron salt, such as FeSO_4_ or Fe_2_(SO_4_)_3_, 0.22 mM) in deionized water was placed in a 16 cm glass tube with internal diameter 1.0 cm and, in the case of argon, purged with argon and stoppered with a rubber septum. The samples for analysis were withdrawn through septum by means of a syringe. In the cases of air and oxygen, these gases were continuously bubbled through the solution. Cuvette was placed into a photochemical reactor and irradiated with six 15 W UV-A lamps for several (typically 10) h. Samples were analyzed in regular time intervals by HPLC-DAD (Agilent, Santa Clara, CA, USA).

#### 3.3.2. Degradation of IPETC under Sunlight

A 100 mL of solution of IPETC (100 ppm, 0.68 mM) and 0.22 mM FeCl_3_ in deionized water was placed in a 500 mL borosilicate glass Erlenmeyer flask and stoppered. The flask was exposed to sunlight for 24 h (morning to morning) in the beginning of July under the cloudless sky at 46° northern latitude. The solution was analyzed by HPLC. 

#### 3.3.3. Reaction with Sodium Hypochlorite

To the 100 mL of the IPETC (1000 ppm, 6.8 mM) solutions in deionized water, buffered with 0.05 M phosphate buffer pH = 6.9, solutions of NaOCl (1.75 M) were added in such amounts, that the ratio NaOCl/IPETC of 2, 4, 6, 8, and 10 mol/mol was obtained. The reaction mixture was allowed to stand 30 min at room temperature and quenched with 2 to 10 mL (proportional to the amount of NaOCl) of 1 M Na_2_SO_3_ solution. After the addition of internal standard (diethylene glycol dimethyl ether), the reaction mixture was extracted with dichloromethane (3 × 5 mL), the organic phase was dried with sodium sulfate and taken for GC analysis.

#### 3.3.4. Reaction with FeSO_4_/H_2_O_2_

To the 100 mL of the IPETC solution in deionized water, iron(II) sulfate and hydrogen peroxide were added in various amounts (i.e., combinations (ppm) 40:200, 40:500, and 100:500, of Fe and H_2_O_2_, respectively) were achieved. The solutions were analyzed by HPLC in regular time intervals.

### 3.4. Syntheses and Isolation of New Compounds

#### 3.4.1. Synthesis of *O*-Isopropyl *N*-ethylcarbamate (**1**)

**Scheme 5:**
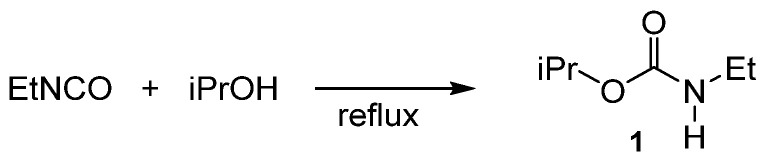
Synthesis of O-isopropyl N-ethylcarbamate (**1**).

Synthesis of *O*-Isopropyl *N*-ethylcarbamate (**1**), presented at Scheme 5.

The compound was synthesized by a modified literature procedure [23]. A mixture of 0.71 g (10 mmol) ethyl isocyanate and 10 mL isopropanol was refluxed for 2 h. The excess isopropanol was evaporated under reduced pressure and 1.23 g (94%) *O*-isopropyl *N*-ethylcarbamate was obtained as a colorless oil. The compound was analyzed by NMR and GC/MS (Agilent, Santa Clara, CA, USA).

^1^H-NMR (CDCl_3_) δ/ppm: 1.13 (t, *J* = 7.2 Hz, 3H), 1.22 (d, *J* = 6.2 Hz, 6H), 3.20 (m, 2H), 4.91 (septet, *J* = 6.2 Hz, 1H). ^13^C-NMR (CDCl_3_) δ/ppm: 15.4 (CH_3_), 22.3 (CH_3_), 35.8 (CH_2_), 67.8 (CH), 156.3 (C).

MS (EI) *m/z* (%): 131 (M^+^, 4), 116 (12), 90 (70), 88 (26), 74 (21), 72 (60), 44 (41), 43 (100), 41 (35), 30 (36).

#### 3.4.2. Isolation of Isopropyl *N*-((ethylimino)(isopropoxy)methyl)-*N*-ethylthionocarbamate (**5**)

A solution of 147 mg (1.00 mmol) of IPETC in 10 mL of 0.05 M phosphate buffer pH = 6.9 was treated with 2.00 mmol of NaOCl (1.1 mL of a 1.77 M solution). After 2 h, the reaction mixture was extracted with dichloromethane (3 × 5 mL), the organic phase dryed with sodium sulfate and concentrated on a rotary evaporator. The mixture was then chromatographed on a column packed with silica gel, using dichloromethane. A 28 mg of pure compound was obtained, which we tentatively assigned, on the basis of spectroscopic data and with the help of advanced NMR measurements, as isopropyl *N*-((ethylimino)(isopropoxy)methyl)-*N*-ethylthionocarbamate (**5**). 

^1^H-NMR (CDCl_3_) δ/ppm: 1.13 (t, *J* = 7.2 Hz, 3H), 1.22 (t, *J* = 7.2 Hz, 3H), 1.25 (d, *J* = 6.2 Hz, 6H) 1.28 (m, 6H), 3.08 (m, 2H), 3.78–3.91 (m, 2H), 4.96 (septet, *J* = 6.2 Hz, 1H), 5.54 (septet, *J* = 6.1 Hz, 1H).

^13^C-NMR (CDCl_3_) δ/ppm: 12.2 (CH_3_), 16.2 (CH_3_), 21.2 (CH_3_), 21.5 (CH_3_), 21.6 (CH_3_), 21.7 (CH_3_), 43.2 (CH_2_), 45.8 (CH_2_), 69.8 (CH), 75.0 (CH), 146.3 (C), 186.7 (C).

MS (EI) *m/z* (%): 260 (M^+^, 1), 217 (22), 175 (90), 147 (13), 104 (23), 88 (13), 72 (71), 44 (68), 43 (100), 41 (40).

HRMS (ESI+) Calcd for C_12_H_25_N_2_O_2_S (MH^+^): 261.1631, found: 261.1634.

## 4. Conclusions

Flotation collector IPETC can be effectively removed from wastewaters by several oxidation methods. Although itself is photochemically inert, it undergoes photooxidation in the presence of iron salts and oxygen. The concentration of oxygen in the reaction mixture has little effect on the rate, since the reoxidation of Fe(II), formed in the oxidation process by molecular oxygen is relatively rapid. Direct sunlight is very efficient in this transformation, under our experimental conditions, the total degradation was achieved in one single day. Iron salts absorb efficiently UV-A light which is abundant in solar radiation and there is no need to use artificial light sources to bring about the photooxidation. As IPETC is used as a flotation collector for sulfide ores, in mine wastewaters containing IPETC the presence of iron compounds is very likely. These waters are usually acidic, which causes iron compounds to be soluble and the photochemical oxidation can take place under sunlight by itself. Similarly, the Fenton reaction system, composed of iron salts and hydrogen peroxide, oxidizes IPETC efficiently, as does sodium hypochlorite in few hours. The oxidation processes are complex, composed of several consecutive and parallel reactions, leading to the formation of simple alcohols, ketones and similar, and, at least in photooxidation to total mineralization. In all three procedures, the main intermediate is *O*-isopropyl *N*-ethylcarbamate which undergoes further decomposition more slowly. Its presence in water might be of concern, since simple carbamates are potential carcinogens.

## Supplementary Materials

Supplementary S1: Mass spectra of compounds 1–5, Supplementary S2: NMR spectra of synthesized compounds 1 and 5, Supplementary S3: Chromatograms of degradation experiments.
