# Supplementary material for: Removal of Flotation Collector O-Isopropyl-N-ethylthionocarbamate from Wastewater
Source: Molecules. 2021 Nov 4;26(21):6676. doi: 10.3390/molecules26216676 (PMC8588165; doi:10.3390/molecules26216676)
Supplement: Supplementary file 1 [file molecules-26-06676-s001.zip › molecules-1402452-supplementary.pdf]

Supplementary S1

MASS SPECTRA OF COMPOUNDS 1-5

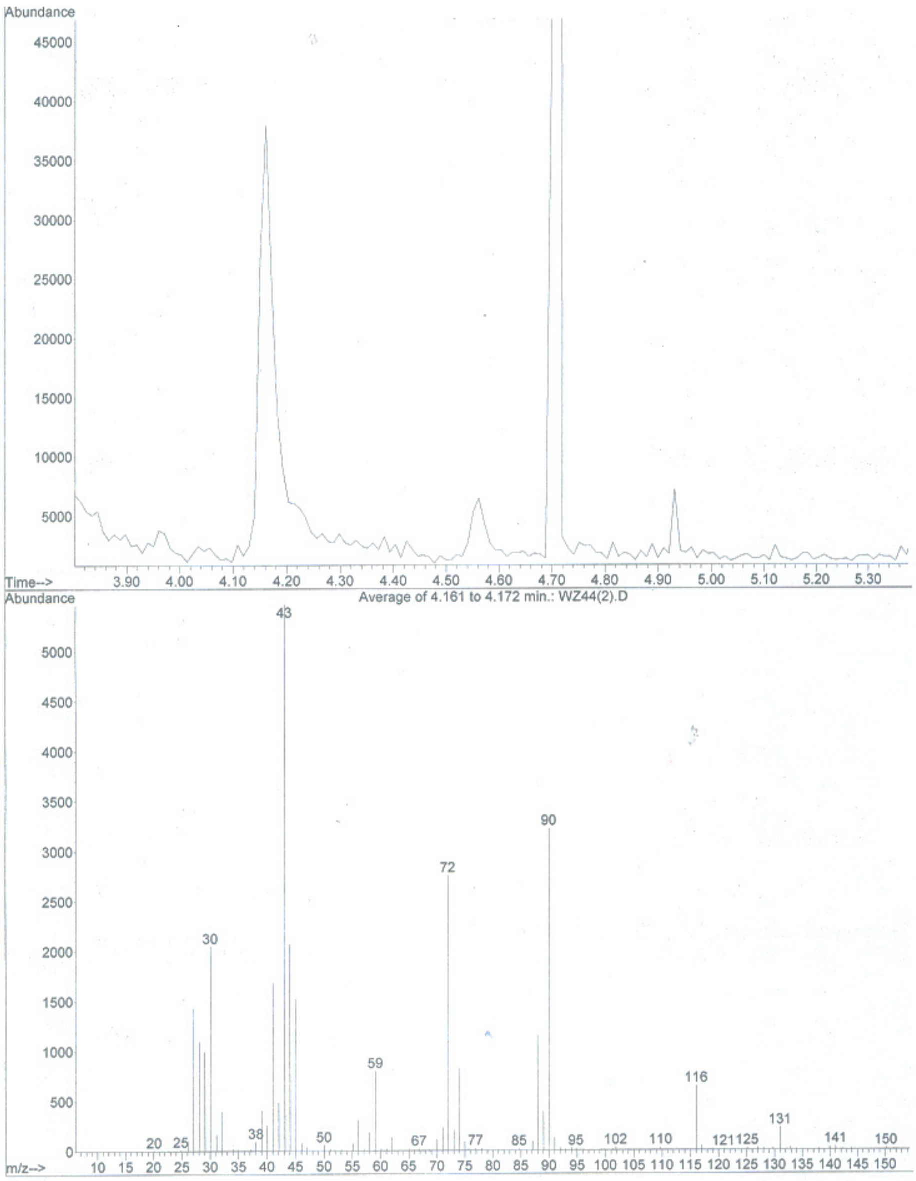

The GC-MS spectrometry of compound 1 (O-Isopropyl N-ethylcarbamate)

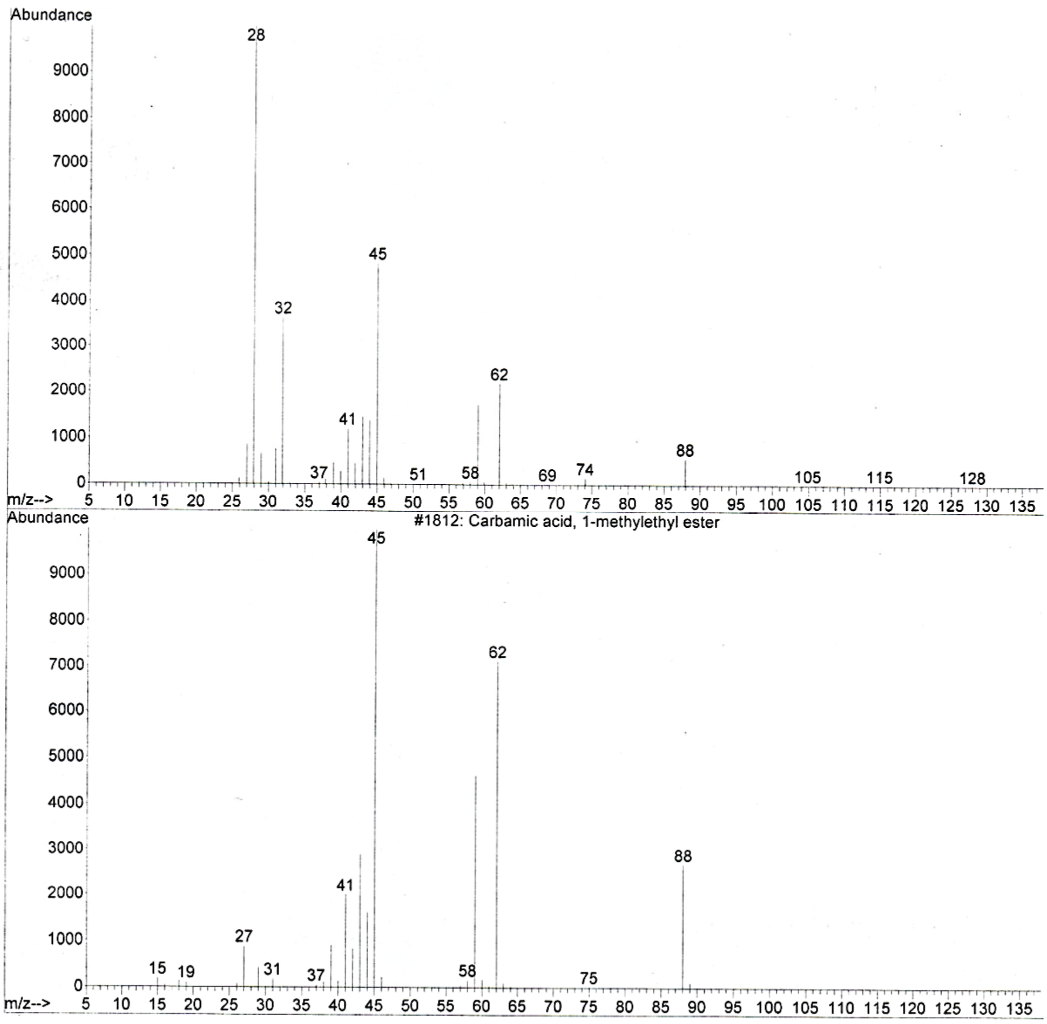

The GC-MS spectrometry of compound 2 (O-isopropyl thionocarbamate)

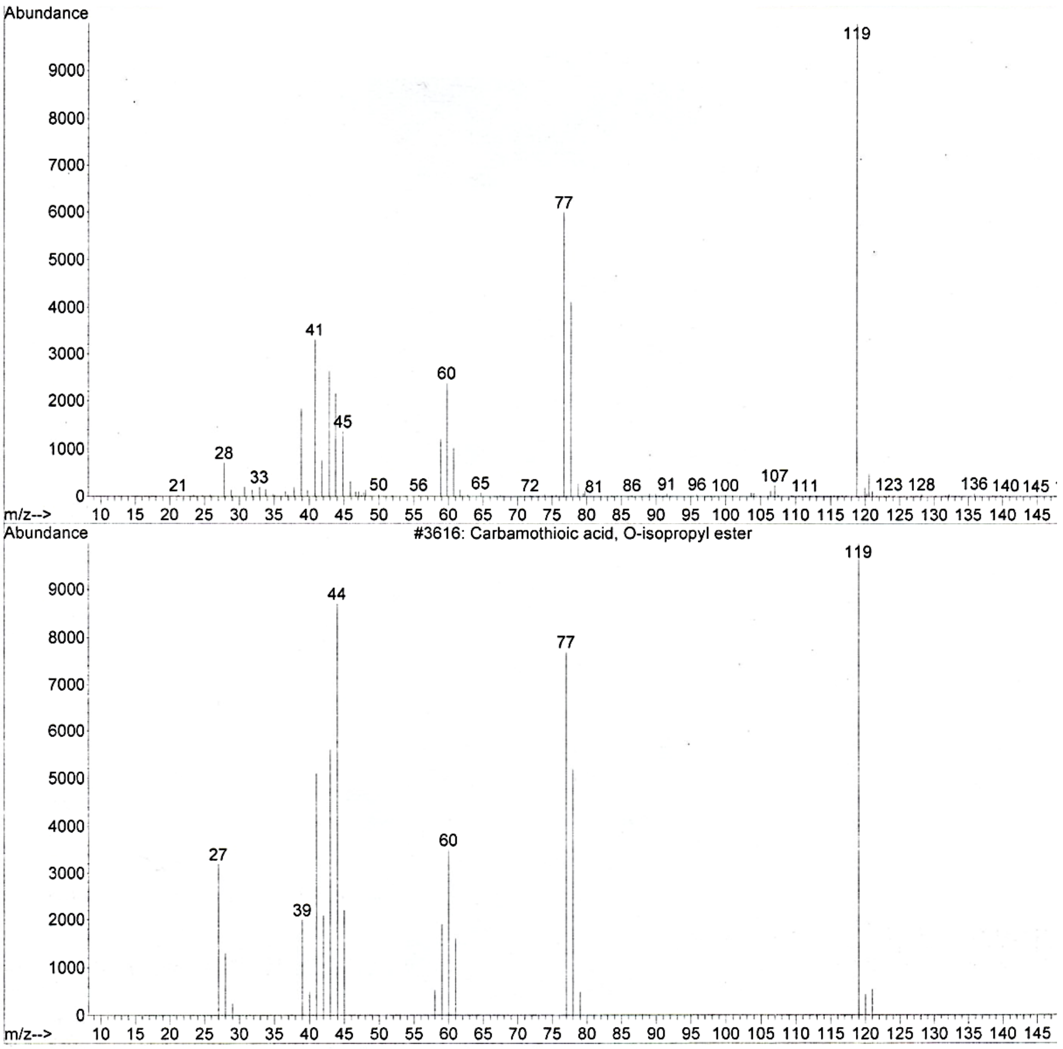

The GC-MS spectrometry of compound 3 (O-isopropyl carbamate)

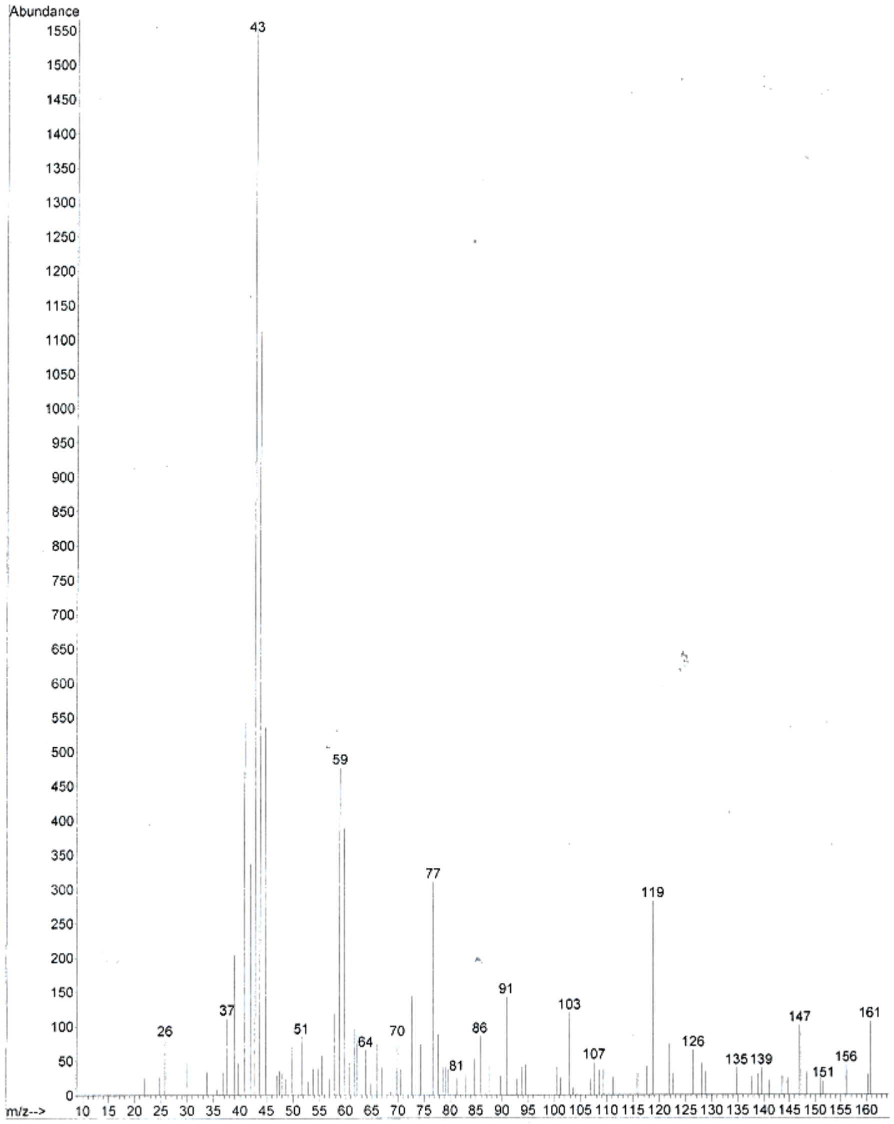

The GC-MS spectrometry of compound 4 (O-isopropyl N-acetylthionocarbamate)

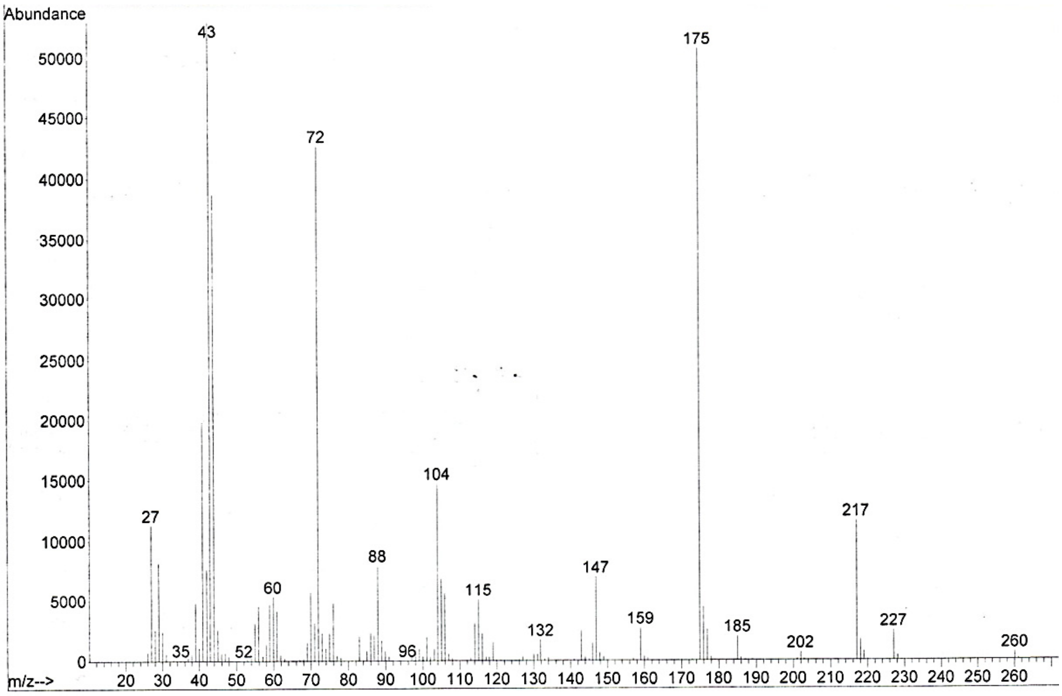

The HPLC-MS spectrometry of compound 5 (isopropyl N-((ethylimino)(isopropoxy)methyl)-N-ethylthionocarbamate)

Supplementary S2

NMR SPECTRA OF SYNTHESIZED COMPOUNDS 1 AND 5

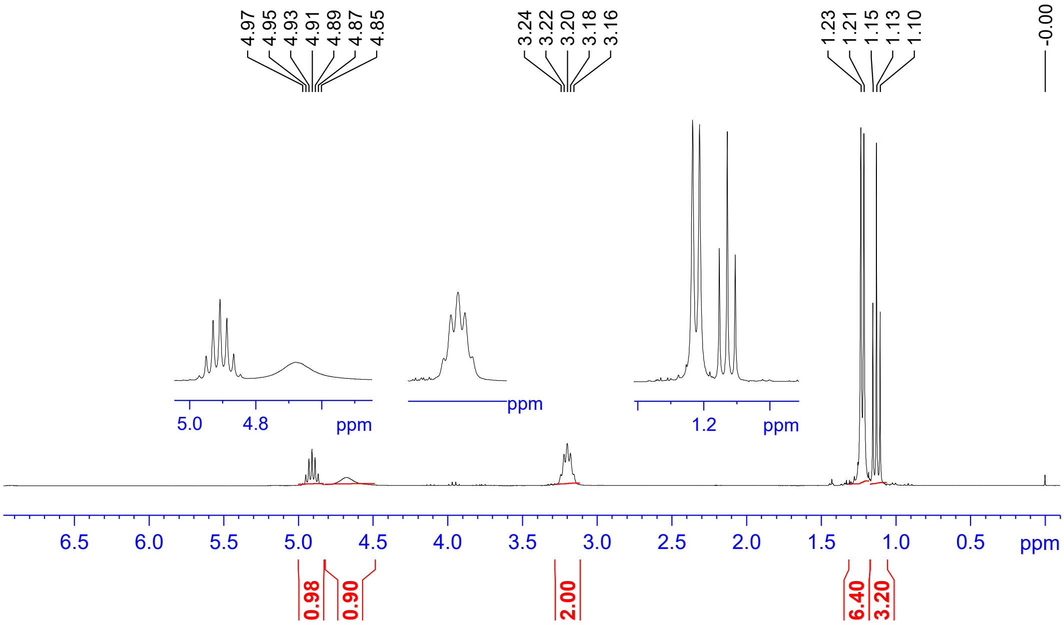

The^{1}H NMR spectrometry of compound 1 (O-Isopropyl N-ethylcarbamate)

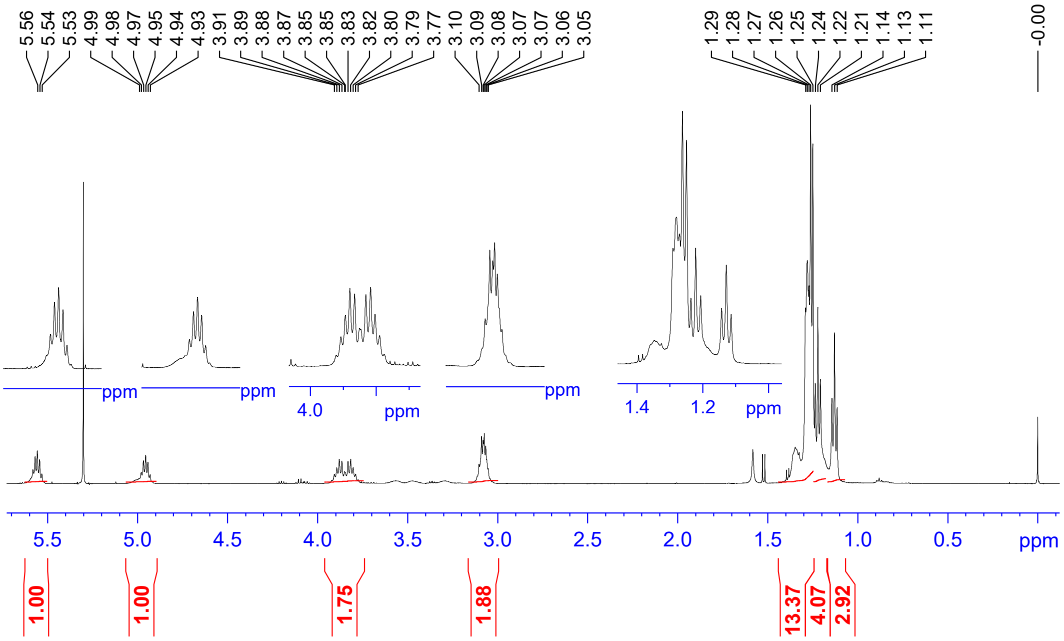

The^{1}H NMR spectrometry of compound 5 (isopropyl N-((ethylimino) (isopropoxy)methyl)-N-ethylthionocarbamate)

Supplementary S3

CHROMATOGRAMS OF DEGRADATION EXPERIMENTS

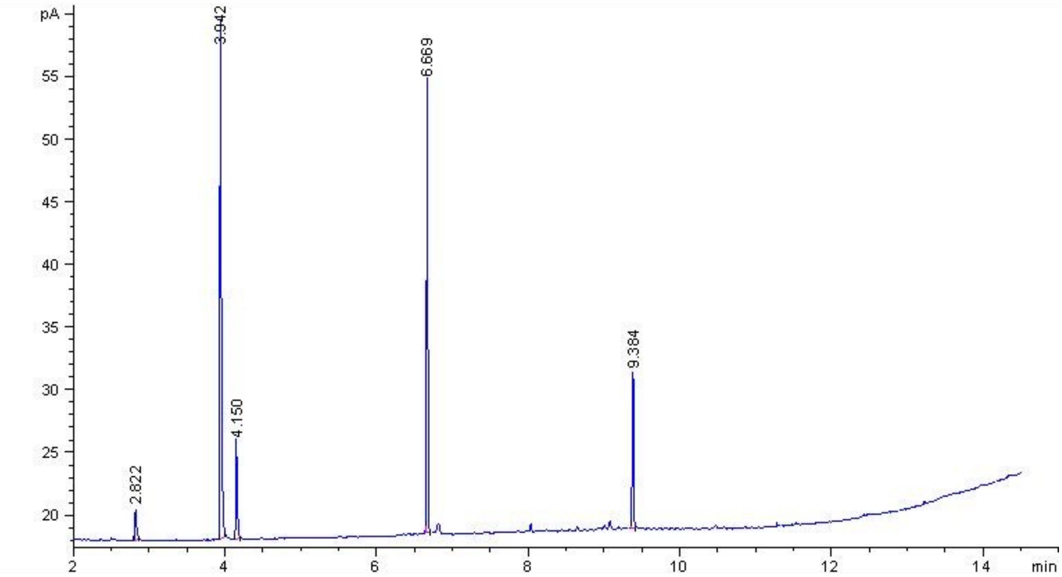

GC chromatogram after reaction with sodium hypochlorite (1 time higher than IPETC molar concentration) for 2 hours

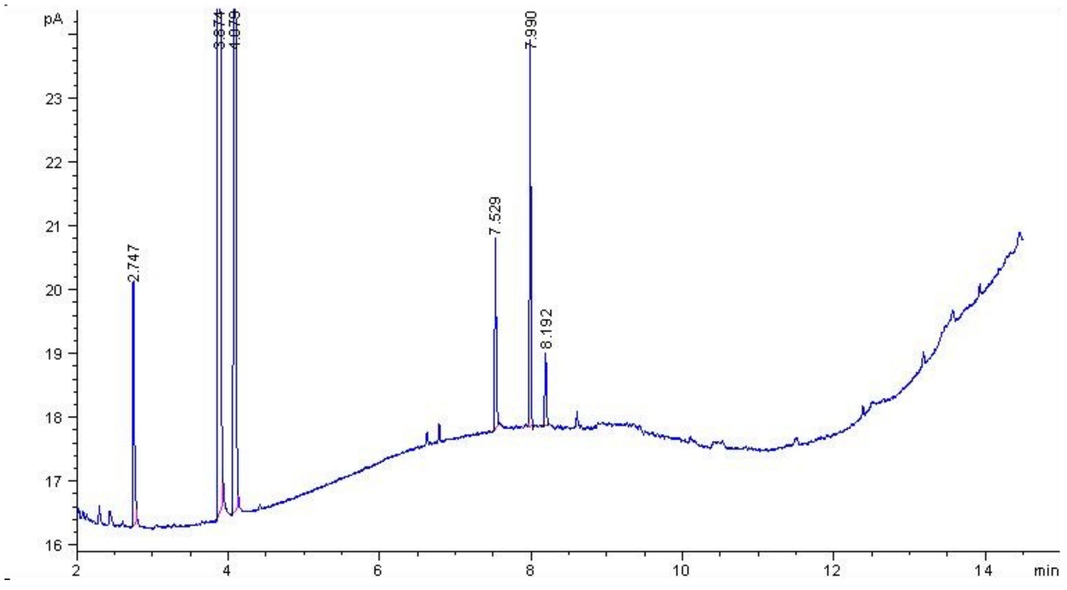

GC chromatogram after reaction with sodium hypochlorite (5 times higher than IPETC molar concentration) for 2 hours

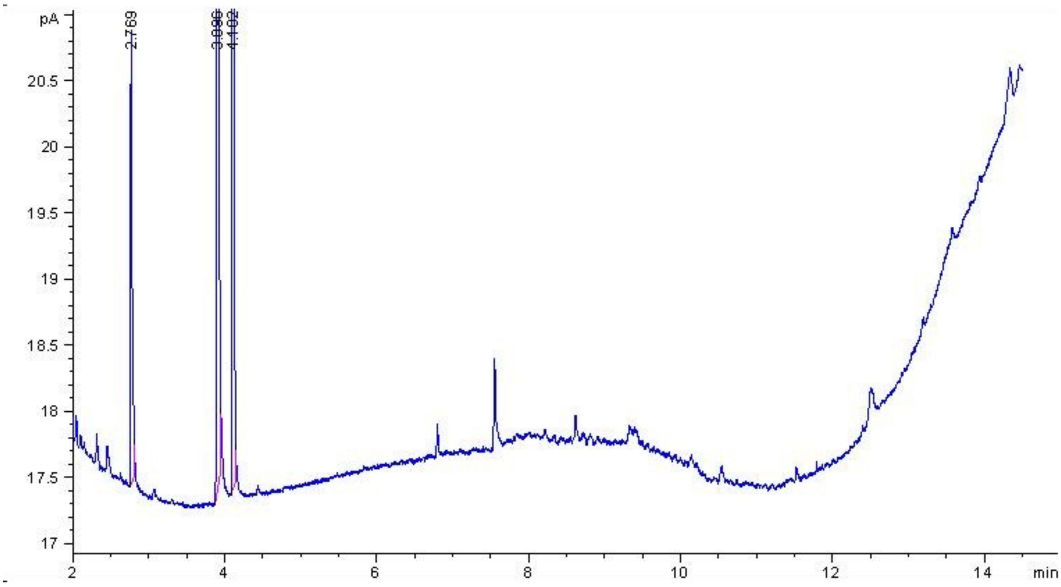

GC chromatogram after reaction with sodium hypochlorite (10 times higher than IPETC molar concentration) for 2 hours

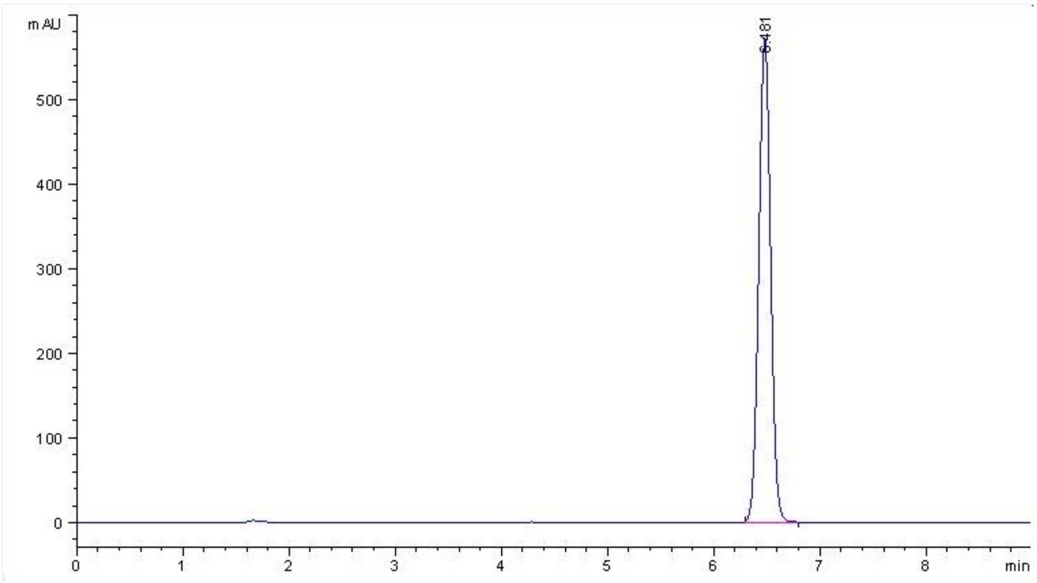

IPETC solution added with iron salt before irradiation (continuous oxygenation)

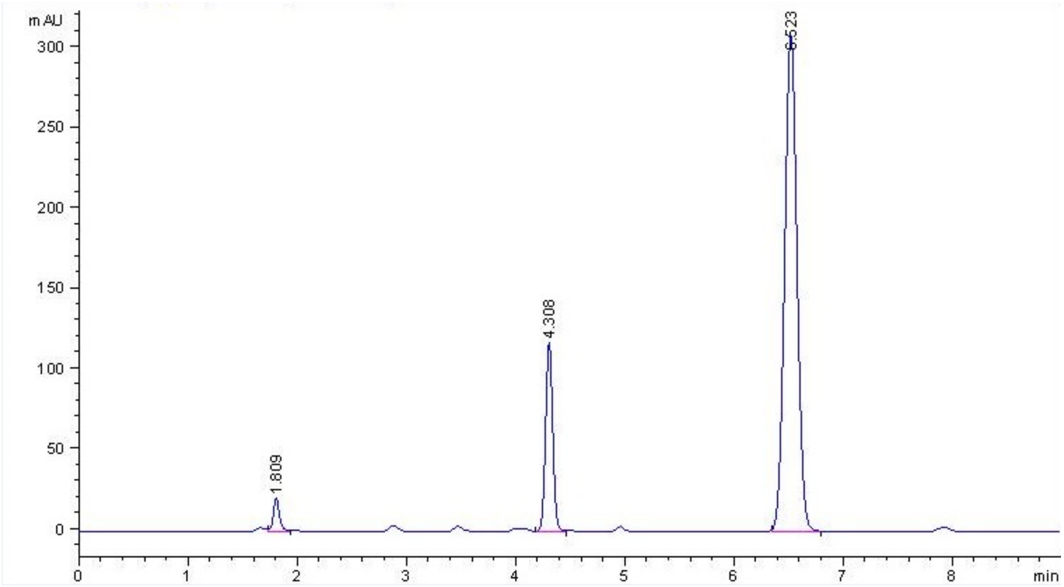

IPETC solution added with iron salt after 2 hours irradiation (continuous oxygenation)

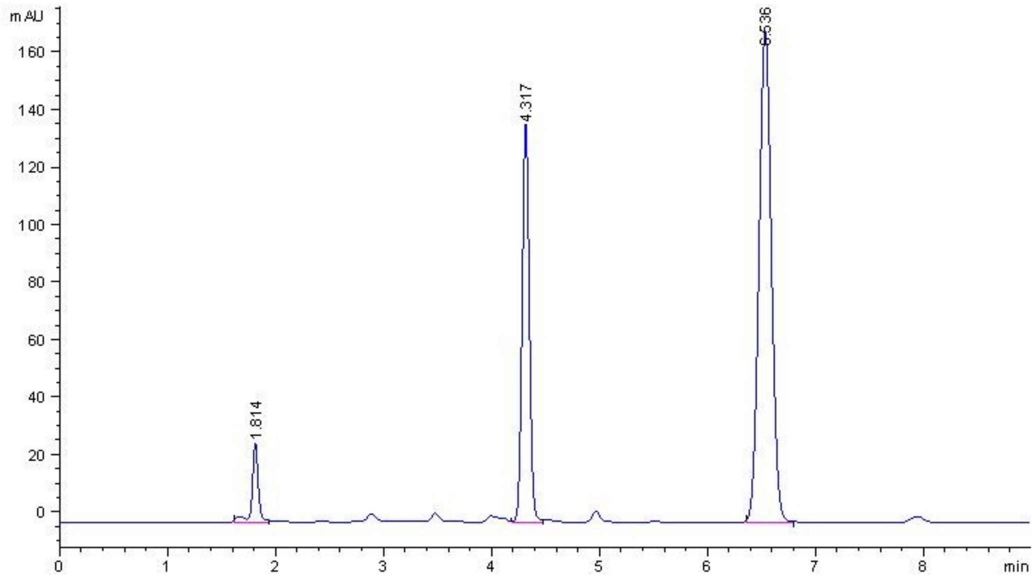

IPETC solution added with iron salt after 4 hours irradiation (continuous oxygenation)

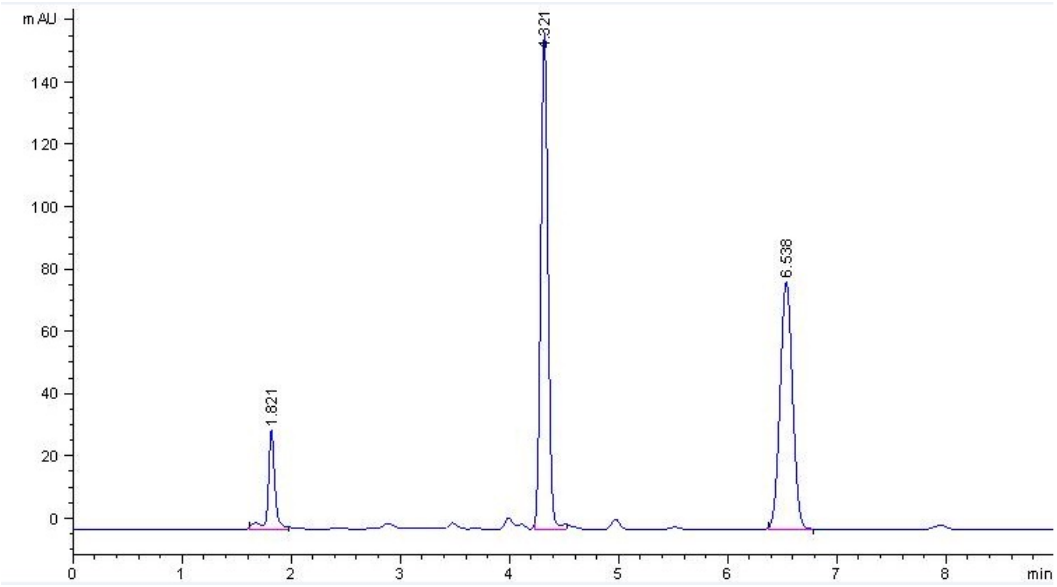

IPETC solution added with iron salt after 6 hours irradiation (continuous oxygenation)

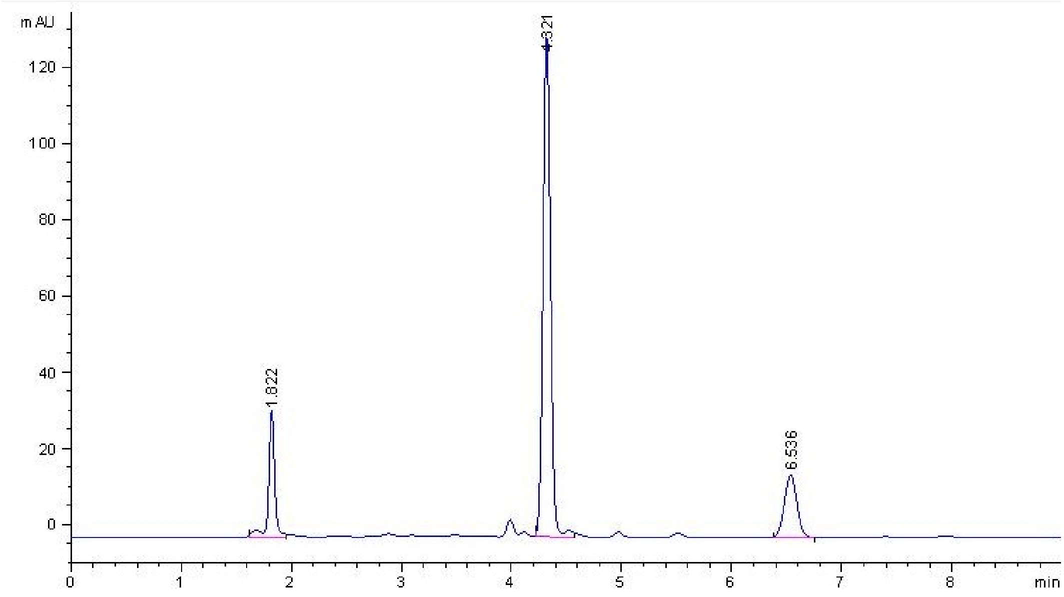

IPETC solution added with iron salt after 8 hours irradiation (continuous oxygenation)

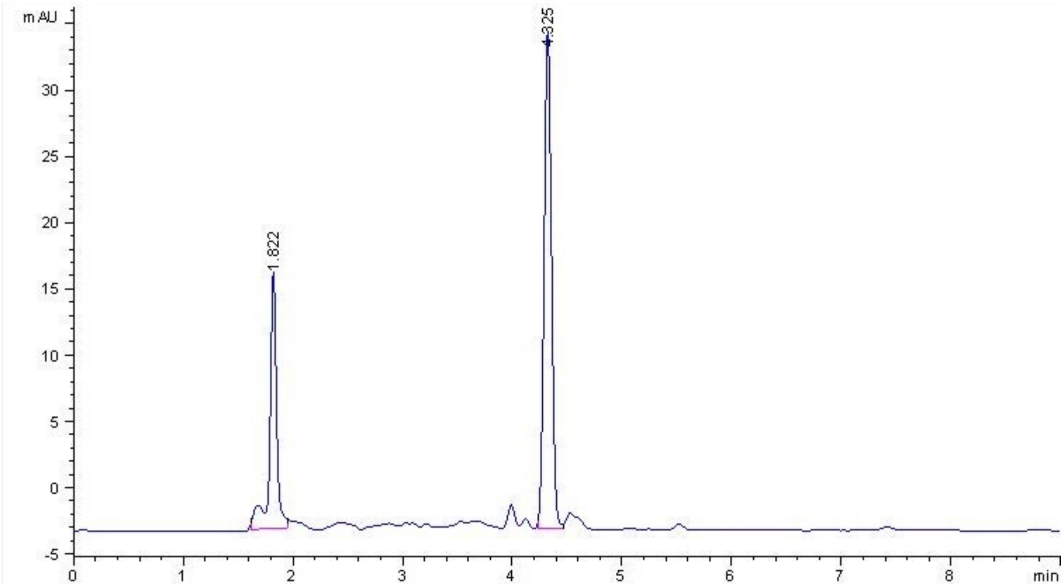

IPETC solution added with iron salt after 10 hours irradiation (continuous oxygenation)

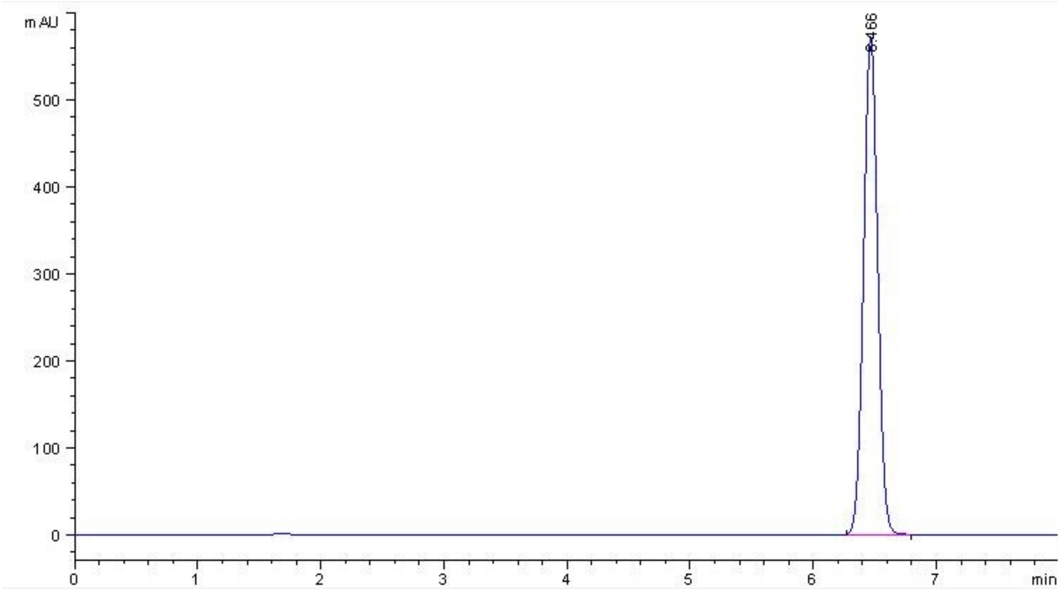

IPETC solution added with iron salt before irradiation (continuous air)

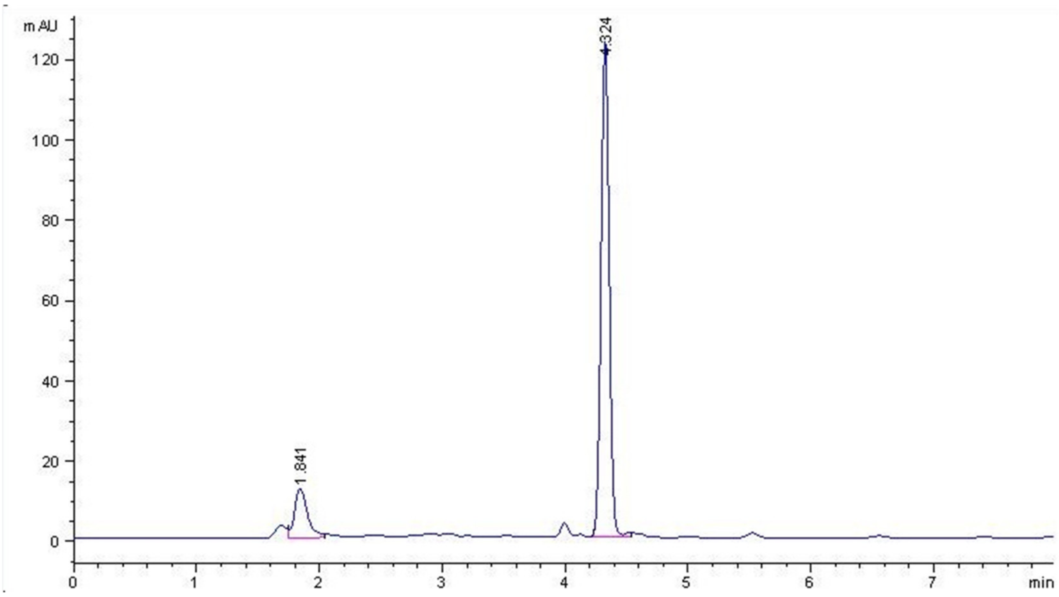

IPETC solution added with iron salt after 10 hours irradiation (continuous air)

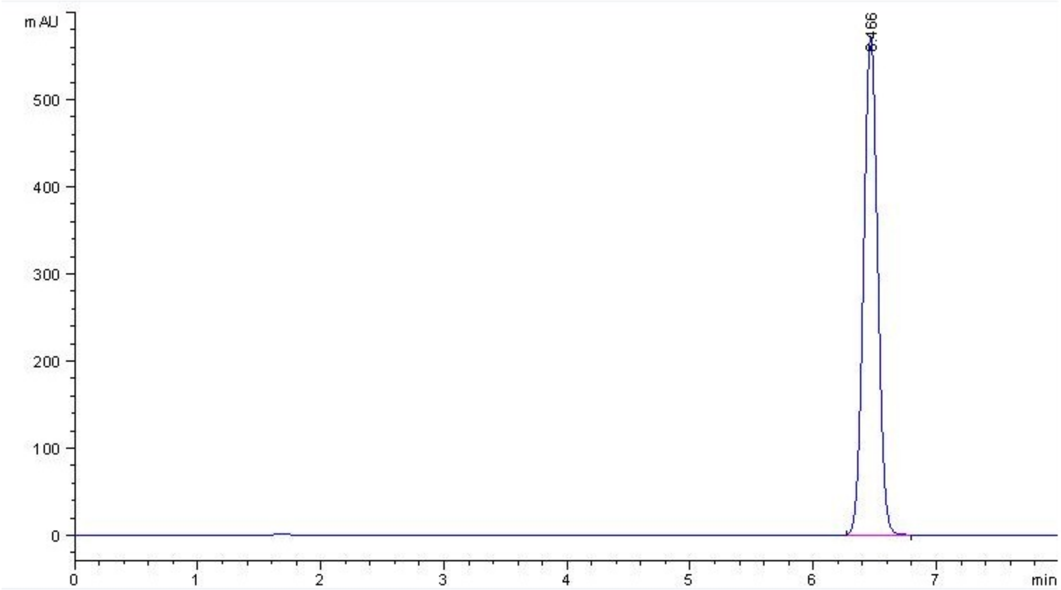

IPETC solution added with iron salt before irradiation (air, not continuous)

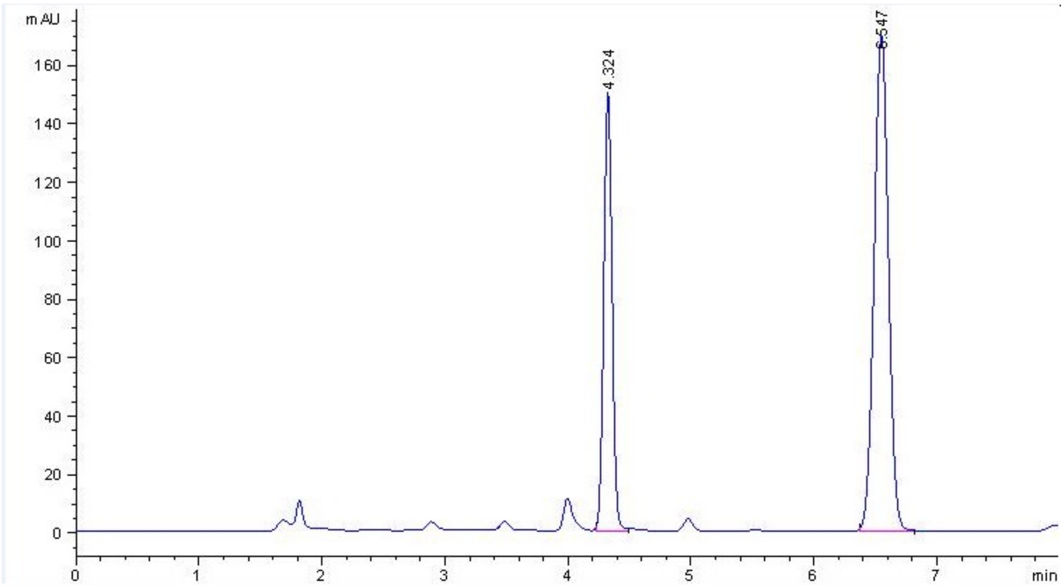

IPETC solution added with iron salt after 10 hours irradiation (air, not continuous)

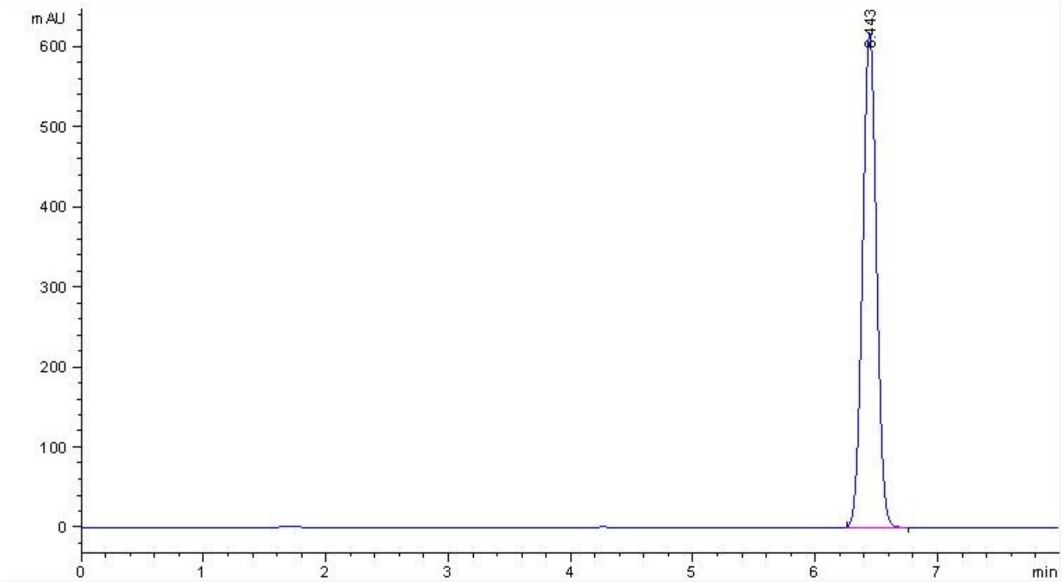

IPETC solution added with iron salt before irradiation (saturated with argon)

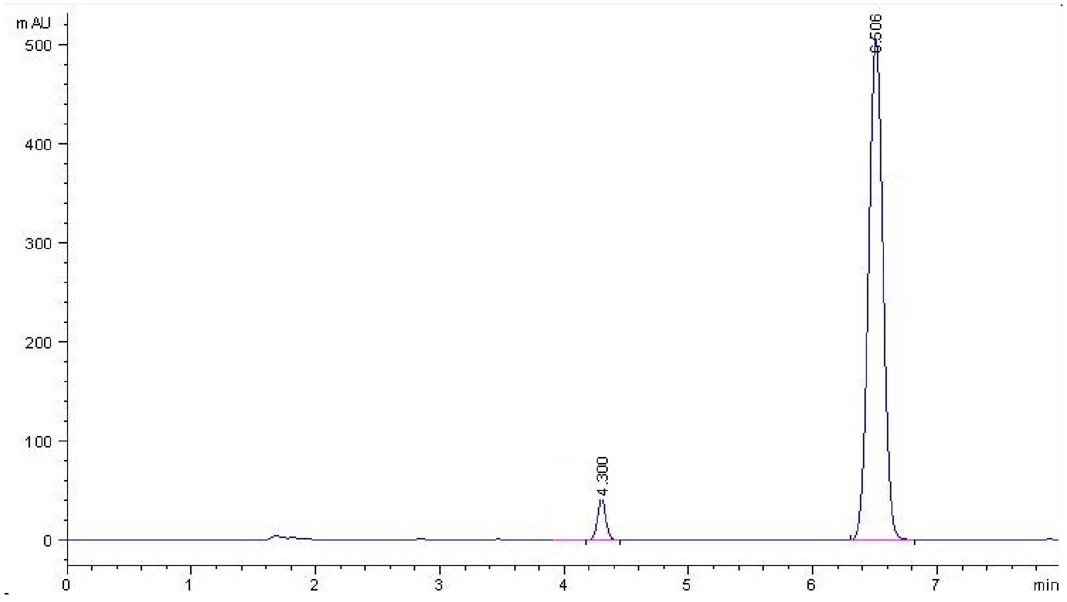

IPETC solution added with iron salt after 10 hours irradiation (saturated with argon)

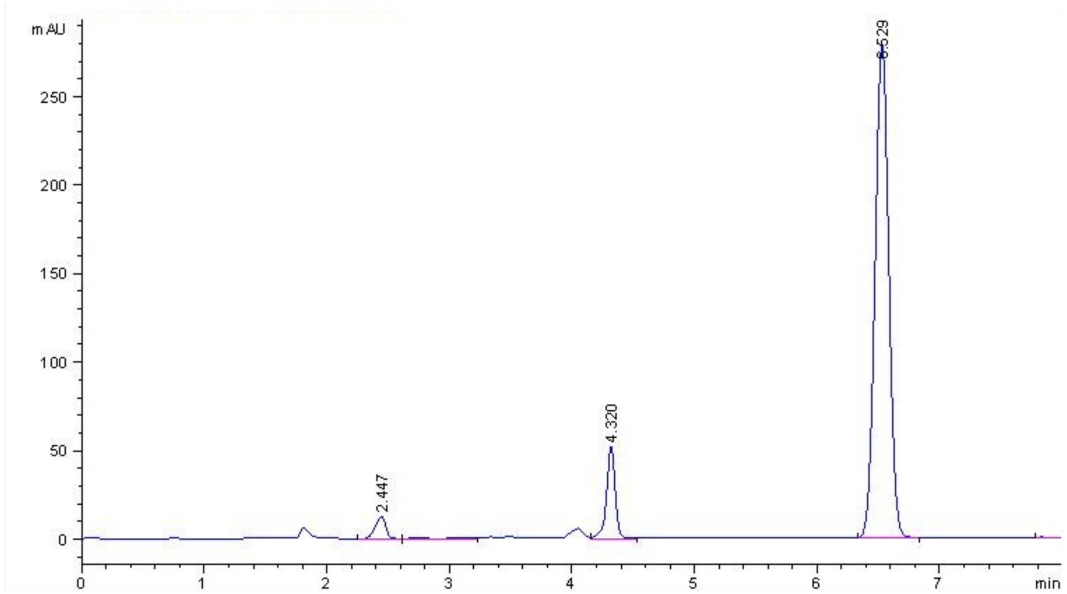

IPETC solution after 10 hours reaction with 40 mg/L Fe^{2+}、200mg/L H_2O_2

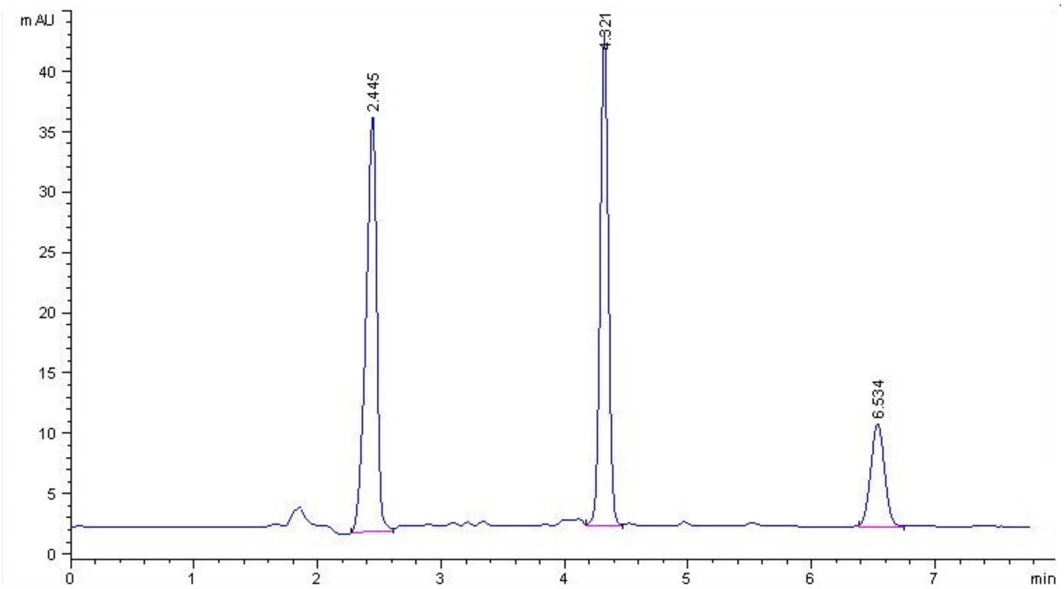

IPETC solution after 10 hours reaction with 40 mg/L Fe^{2+}、500mg/L H_2O_2

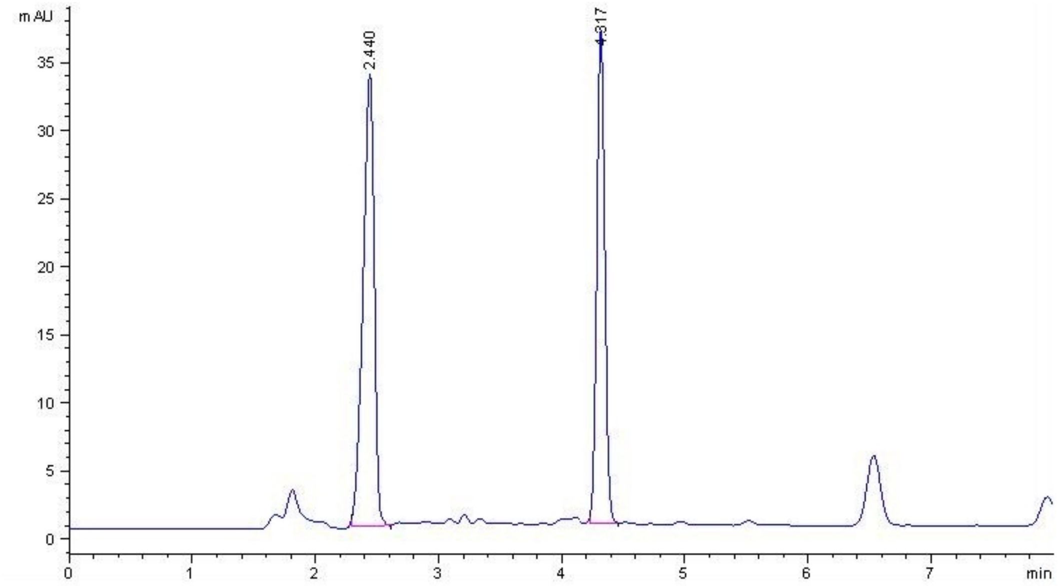

IPETC solution after 10 hours reaction with 100 mg/L Fe^{2+}、500mg/L H_2O_2
